# Dropsy of the Os Uteri

**Published:** 1843-05

**Authors:** 


*Dropsy of the Os Uteri.*—Under this name Jobert describes tumefaction of the mouth and neck of the uterus, which most frequently occurs among women of a lymphatic temperament, being, according to his observations, confined to those who have never borne a child, and who menstruate but feebly. Examined by the aid of the speculum, the os uteri is seen to be so much swollen as almost wholly to conceal the orifice, and it gives on pressure a sense of fluctuation. It is uniformly pale and flabby, and may sometimes be ulcerated, but it is not in general organically diseased. On carefully introducing a bougie through the orifice, a quantity of transparent, flocky, light-colored fluid usually escapes from the cavity of the uterus; and, at the same time, the neck and mouth become relieved of a portion of their tumefaction. This event may happen consequent on a spontaneous discharge of the fluid, and always attends more or less the recurrence of the menstral discharge. The cause of the affection has been attributed to a distension and superabundant secretion of the glandular follicles of the neck and mouth of uterus. For its treatment after the evacuation of the contents of the uterus, Jobert advises free incisions to be made in the os uteri, in the direction from centre to circumference (*dans le sens des commissures*). The granulation of the wounds thus made, produces concurrently, as he says, an enlargement of the orifice of the uterus, which effectually obviates a return of the disease.—*Lon. Lancet.*

